# Llanfairfechan Convalescent Home

**Published:** 1887-07-30

**Authors:** Anna M. Butler

**Affiliations:** Sister-in-Charge


### LANFAIRFECHAN CONVALESCENT HOME.

Will you allow me to ask those of your readers who have enjoyed the lovely mountain scenery of North Wales, and others who sympathise with Convalescent work, to contribute towards the Building Fund of the above Home? We require £3,000 to enable us to make use of a splendid site given to us by a generous landowner, on condition we can raise the necessary funds. So much good has been done already, that we hope to continue the work in a house built according to our requirements, free from rent. Contributions will be thankfully received by Miss Butler at the Home, or by Messrs. Williams and Co., Old Bank.—I am, Sir, yours faithfully,

Anna M. Butler,
Sister-in-Charge.

